# Mesenchymal Stem Cell Therapy for the Treatment of Vocal Fold Scarring: A Systematic Review of Preclinical Studies

**DOI:** 10.1371/journal.pone.0162349

**Published:** 2016-09-15

**Authors:** Vibe Lindeblad Wingstrand, Christian Grønhøj Larsen, David H. Jensen, Kristian Bork, Lars Sebbesen, Jesper Balle, Anne Fischer-Nielsen, Christian von Buchwald

**Affiliations:** 1 Department of Otorhinolaryngology, Head and Neck Surgery and Audiology, Rigshospitalet, University of Copenhagen, Copenhagen, Denmark; 2 Cell Therapy Facility, Blood Bank, Department of Clinical Immunology, Copenhagen University Hospital Rigshospitalet, Copenhagen, Denmark; Wake Forest Institute for Regenerative Medicine, UNITED STATES

## Abstract

**Objectives:**

Therapy with mesenchymal stem cells exhibits potential for the development of novel interventions for many diseases and injuries. The use of mesenchymal stem cells in regenerative therapy for vocal fold scarring exhibited promising results to reduce stiffness and enhance the biomechanical properties of injured vocal folds. This study evaluated the biomechanical effects of mesenchymal stem cell therapy for the treatment of vocal fold scarring.

**Data Sources:**

PubMed, Embase, the Cochrane Library and Google Scholar were searched.

**Methods:**

Controlled studies that assessed the biomechanical effects of mesenchymal stem cell therapy for the treatment of vocal fold scarring were included. Primary outcomes were viscoelastic properties and mucosal wave amplitude.

**Results:**

Seven preclinical animal studies (n = 152 single vocal folds) were eligible for inclusion. Evaluation of viscoelastic parameters revealed a decreased dynamic viscosity (η’) and elastic modulus (G’), i.e., decreased resistance and stiffness, in scarred vocal folds treated with mesenchymal stem cells compared to non-treated scarred vocal folds. Mucosal wave amplitude was increased in scarred vocal folds treated with mesenchymal stem cells vs. non-treated scarred vocal folds.

**Conclusion:**

The results from these studies suggest an increased regenerative effect of therapy with mesenchymal stem cells for scarred vocal folds and are encouraging for further clinical studies.

## Introduction

Parts of normal vocal fold (VF) tissue are often replaced with scar tissue during the healing process after trauma, inflammatory disorders, iatrogenic lesions after surgery, and as a well-known side-effect of the treatment of head and neck cancers with radiotherapy.[1]

The tissue in the wound healing process after any type of insult may be replaced by fibrous scar tissue, which primarily consists of an excessive, disorganized extracellular matrix. The scar tissue matrix is generally stiffer than the extracellular matrix it replaces. This matrix is of particular concern in areas surrounding and including the vocal folds because the normal functions of these tissues depend on their elastic and vibratory properties.

True VFs are one of the most important structures used during normal phonation because of their unique anatomic structure, which is essential for regulating the phonatory output.[2] The mucosa is of particular interest because it is the most common site for injury.[3] Proteins such as elastin, which is highly abundant in the elastic cone, and other extra cellular matrix (ECM) proteins create a specialized pliable connective tissue that supports the viscoelasticity of the VFs during vibration.[4] Diminished elasticity of the vocal folds reduces the ability to phonate normally, which the patient will experience as a strained, weakened or hoarse voice. The inability to phonate normally decreases both psychosocial and physical well-being.[3] Increased mucosal stiffness is measured as a decreased dynamic viscosity and elastic modulus using rheometry. Changes in the extracellular matrix, such as the change from highly organized elastin to disorganized Type I collagen, primarily explain the change in phonation.[5] The altered composition of ECM proteins is observed microscopically and correlates with macroscopically altered biomechanical properties, which also lead to changes in the mucosal waves during phonation.[6–8] Notably, these altered biomechanical properties predict the quality of phonation.[6]

However, the surgical and medical treatments to ameliorate the reduced functions of the vocal folds after scarring produce limited effects. Therefore, the potential for regenerative therapy using stem cells is of growing interest in the field.

Mesenchymal stem cells (MSCs), which were first described in the bone marrow to support hematopoiesis,[9] are so-called adult stem cells that give rise to all cell types of mesodermal origin, such as osteocytes, chondrocytes and fibroblasts.[10] MSCs are isolated from a wide variety of tissues, but these cells are most often harvested from adipose tissue (adipose-derived MSCs, ASCs, AdMSCs) or bone marrow (bone marrow-derived MSCs, BM-MSCs).[11] MSCs have received much attention over the past several decades, especially because of their immunomodulatory, regenerative and trophic properties, but also because these cells are easily harvested and expanded, the latter being a necessary function to obtain sufficient numbers of cells.[12, 13] The exact mechanism of action is not known, but various models of the therapeutic effects of MSCs have been proposed. [12]

Several clinical trials with MSCs in various fields of regenerative medicine were performed, and promising results with minimal side effects of the treatment were demonstrated.[12, 14–16]

Some studies used non-expanded cells from the fat-derived stromal vascular fraction (SVF), which included a heterogeneous cell population of endothelial, hematopoietic and pericytic origin and only a minor fraction of adipose stem and precursor cells.[17]

Several types of therapies were evaluated to ameliorate the effects of scar tissue formation in the VF, such as implantation of scaffolds and injection of growth factors.[18] No clinical trial using MSCs for treating scar tissue formation on humans has been published. Several animal studies have evaluated the effects of MSC therapy in reducing VF scarring. These studies include evaluations of macroscopic scar formation and morphology, histological changes, transcriptional upregulations, stem cell detection and functional outcomes. A comprehensive list of all outcome measures from the preclinical studies may be found in S1 Table. The direct correlation between the morphological, histological, and transcriptional changes and stem cell persistence and the regenerative effect of the VFs cannot be performed, largely because of the unknown mechanism of action of these stem cells. Therefore, we chose to evaluate the biomechanical changes, which provide a more direct evaluation of VF function. This study systematically evaluated the literature on animal models concerning the biomechanical properties and vibration patterns of scarred vocal folds after being injected with MSCs to evaluate their potential in a future human trial.

## Materials and Methods

### Inclusion criteria

Controlled animal studies that evaluated MSCs as a treatment for vocal fold scarring and included biomechanical measures were eligible for inclusion, regardless of the publication date. Only publications in English were included. Unpublished clinical trials were accessed via ClinicalTrials.gov. The outcome variables of interest were any type of viscoelastic or mucosal wave measurement of the VFs after MSC treatment.

### Search strategy

An electronic search was performed on PubMed, Embase, the Cochrane Library and Google Scholar. The following keywords (MeSH terms included) were used: vocal folds or vocal cords and stem cell or MSC or mesenchymal stem cell or processed lipoaspirate cell or stromal vascular fraction cell. The Search strategy appendix in S1 Appendix provides a detailed description of the search strategy. The date of the last search was 1 April 2016.

The following data were extracted from the included studies: study design, randomization, blinding, study participants, graft donor, source of graft, intervention, control groups, persistence of the stem cell, statistical tests, biomechanical method, time after stem cell injection and results from measurements of dynamic viscosity, elastic modulus and amplitude.

## Results

**Fig 1:**
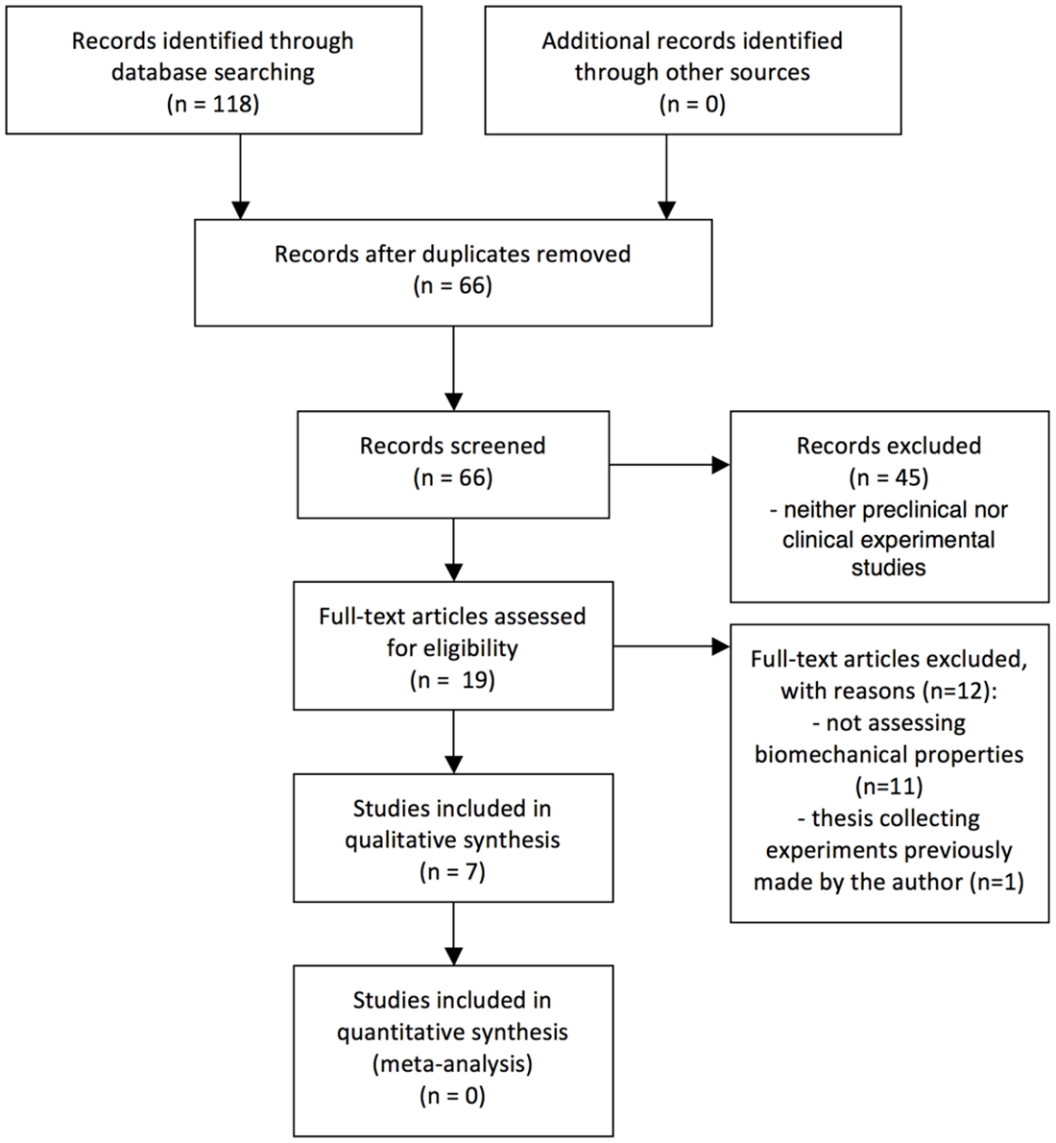
PRISMA chart.

The electronic searches identified 118 potentially eligible studies, only seven of which [19–25] met the inclusion criteria (Fig 1). These seven studies, which addressed 152 single VFs in 76 animals, were included in the qualitative analysis. Tables 1 and 2 summarizes the interventions and study designs. All studies were controlled animal studies. One study reported a blinded evaluation of the results, and three studies reported randomization of animals into the intervention or control group. Six of the studies examined rabbit vocal folds, and one study investigated vocal folds in dogs. Bone-marrow derived MSCs were used in six studies, and one study used adipose-derived MSCs. Three studies examined a combined approach with MSCs and small intestinal submucosa, hyaluronic acid/cross-linked alginate hydrogel or an atelocollagen sponge. The stem cells were injected directly into the injured area of the vocal fold in six studies, and the cells were implanted in combination with an atelocollagen sponge at the injured site in one study. Only one study used autologous MSCs, and five studies used xenotransplanted MCSs from another animal or human MSCs. One study used allogeneic MSCs. The immunosuppressive drug tacrolimus was injected into the animals in three studies to minimize the risk of rejection of the injected stem cells.

**Table 1:** Overview of studies.

Author (year) [reference]	Study design	Animal (VFs in control and intervention)	Graft donor source	Origin of MSCs	Intervention(s)	Disease model of scarring	Control group(s)
Choi et al (2014) [19]	Controlled trial	Rabbits (24 rabbits were included, with bilateral scarring of VFs. The control was the left VF, leaving 8 VFs per intervention)	Rabbit MSCs and SIS from pig	Bone marrow	Injection of 2x10^7^ MSCs mixed with SIS powder Injection with 2x10^7^ MSCs Injection with SIS powder	Scarring of vocal folds with electrocoagulator	A group with scarring without interventions
Hertegård et al (2006) [20]	Controlled trial	Rabbits (10 rabbits were included of which 12 VFs were scarred and 8 were left unscarred)	Human	Bone marrow	Injection with 8x10^4^ MSCs. The MSC treated animals were also given tacrolimus Injection with saline	Scarring of vocal folds with localized excision of the mucosa and superficial thyroarytenoid muscle with micro scissors	A group with saline injection of scarred vocal folds A group without scarring that were left untreated
Kim et al (2013) [21]	Randomized, controlled trial	Rabbits (24 rabbits were included, 8 were uninjured, 4 were unilaterally scared and 4 were bilaterally scarred leaving 8 animals unaccounted for)	Mice	Bone marrow	Injection of 1x10^5^ MSCs Injection of PBS	Excision of the VF epithelium and lamina propria with micro cup forceps	A group without scarring of VFs A group with scarred vocal folds treated with PBS
Kim et al (2014) [22]	Randomized, controlled, blinded trial	Rabbits (40 animals in total, 8 per group, leaving 16 VFs per group)	Human	Adipose tissue	Injection of 1x10^6^ MSCs Injection with 1x10^6^ MSCs and HA/ALG Injection with PBS Injection with HA/ALG	Excision of the VF epithelium and lamina propria using microsurgical instruments	A group without scarring A group with scarred VFs treated with PBS
Ohno et al (2011) [23]	Randomized, controlled trial	Dogs (12 dogs, with 8 receiving bilateral scarring of VFs, and 4 with unilateral scarring)	Dog MSCs and human atelocollagen	Bone marrow	Implantation of 1x10^6^ MSCs in an atelocollagen sponge Implantation of atelocollagen sponge	Stripping of the epithelium and lamina propria to the thyroarytenoid muscle with micro scissors and micro forceps	A group with no scarring
Svensson et al (2010) [24]	Controlled trial	Rabbits (11 rabbits, where eighteen VFs were scarred and 4 were left unscarred)	Human	Bone marrow	Injection of 0.8-1x10^5^ MSCs and afterwards tacrolimus Injection of saline	Excision of the mucosa and superficial layer of the thyroarytenoid muscle with micro cup forceps	A group with saline injection in scarred vocal folds A group without scarring
Svensson et al (2011) [25]	Controlled trial	Rabbits (12 rabbits in total, of which 20 VFs were scarred and 4 VFs were unscarred controls	Human	Bone-marrow	Injection of 0.8-1x105 MSCs and afterwards tacrolimus. Saline	Excision of the mucosa and superficial layer of the thyroarytenoid muscle with micro cup forceps	An unscarred group A scarred group treated with saline

VFs, vocal, MSC, Mesenchymal stem cell, SIS, small intestine submucosa, PBS, phosphate-buffered saline, HA, Hyaluronic acid, ALG, mildly cross-linked alginate hydrogel

**Table 2:** Overview of studies (continued).

Author (year) [reference]	Intervention(s)	Persistence of cells	Statistical tests
Choi et al (2014) [19]	Injection of 2x10^7^ MSCs mixed with SIS powder Injection with 2x10^7^ MSCs Injection with SIS powder	> 8 weeks > 8 weeks	One way analysis of variance (ANOVA): analysis between comparative groups Tukey-HSD test: difference between mean values Coefficient of correlation test: analysis of mean value Student T-test: analysis of mean value
Hertegård et al (2006) [20]	Injection with 8x10^4^ MSCs. The MSC treated animals were also given tacrolimus Injection with saline	At 4 weeks; 0,18 engraftment	Nonparametric comparisons between the groups Wilcoxon: comparison of mean values
Kim et al (2013) [21]	Injection of 1x10^5^ MSCs njection of PBS	> 4 weeks	Mann-Whitney test: differences between two groups Kruskal-Wallis test + post hoc - - Dunn’s test: comparison of the values among the tree groups
Kim et al (2014) [22]	Injection of 1x10^6^ MSCs Injection with 1x10^6^ MSCs and HA/ALG Injection with PBSInjection with HA/ALG	> 1 month > 1 month	The Mann-Whitney test: significance of differences between two groups Kruskal-Wallis test + Dunns’ post hoc test: comparison of the three groups Bonferroni’s post hoc test: analysis of rheological data using two-way analysis of variance
Ohno et al (2011) [23]	Implantation of 1x10^6^ MSCs in an atelocollagen sponge Implantation of atelocollagen sponge	Not measured	Paired r-test: differences in the NMWA Welch's i-test: differences in CR-NMWA
Svensson et al (2010) [24]	Injection of 0.8-1x10^5^ MSCs and afterwards tacrolimus Injection of saline	At 3 months: no detectable MSCs	Mann-Whitney U test: nonparametric comparisons
Svensson et al (2011) [25]	Injection of 0.8-1x105 MSCs and afterwards tacrolimus Saline	At 10 weeks: no detectable MSCs	Mann–Whitney U test: differences between two groups Binomial test: differences between the three groups F test: regressions analysis

The species, source of the stem cell graft, evaluation time, disease model, intervention and statistical tests varied significantly across the studies, and a meta-analysis would be meaningless.

### Biomechanical outcomes after treatment of VF scarring with MSCs

Five of the studies reported biomechanical measures, e.g., dynamic viscosity and elastic modulus, after the induction of VF scarring. Table 3 describes these results. Two study groups performed these five studies: [20, 24, 25] and [21, 22]. Notably, the cell dose in reference [22] differed 10-fold from the other studies.

**Table 3:** Study results from rheometry.

Testing method	Author (year) [reference]	Time from wounding to intervention	Time from intervention to biomechanical testing	Dynamic viscosity comparisons in scarred groups*	P—value	Elastic modulus comparisons in scarred groups*	P—value
Parallel plate rheometry	Hertegård et al (2006) [20]	0 days	4 weeks	Saline vs. MSC injection	P = 0.07	Saline versus MSC injection	P < 0.01
Parallel plate rheometry	Svensson et al (2010) [24]	0 days	3 months	Saline vs. MSC injection	P < 0.05	Saline versus MSC injection	P < 0.05
Parallel plate rheometry	Svensson et al (2011) [25]	9 weeks	10 weeks	Saline vs. MSC	P = 0.03	Saline versus MSC injection	P < 0.001
Parallel plate rheometry	Kim et al (2013) [21]	0 days	1–3 months	PBS vs. MSC	P = 0.2	PBS versus MSC	P = 0.34
Parallel plate rheometry	Kim et al (2014) [22]	0 days	1 and 3 months	PBS vs. MSC PBS vs. MSC + HA/ALG	Not reported P < 0.01	PBS versus MSC PBS versus MSC + HA/ALG	Not reported P < 0.01

* Only comparisons deemed relevant are reported.

Two of these five studies reported a statistically significant improvement in dynamic viscosity in the MSC-treated group versus a saline-treated control group. The difference in dynamic viscosity was only marginally significant in one study (P = 0.07), but it was insignificant in the other study (P = 0.2). The last study did not report a comparison between the MSC-treated and saline control groups.

Five studies compared elastic modulus measurements in an MSC-treated group versus a saline-treated control group. Three of these studies demonstrated a significant improvement in elastic modulus in the MSC-treated group versus the saline control group, but one study did not demonstrate an improvement (P = 0.34). The last study did not report the statistics of this comparison.

None of the studies reported a measure of the magnitude of the difference, such as a fold-change or percentage difference, in dynamic viscosity or elastic modulus in the MSC-treated versus saline control group. Two of the studies that compared the viscoelastic results in an MSC-treated group versus a normal control group that was not VF scarred demonstrated that neither the elastic modulus nor the dynamic viscosity between the scarred VFs treated with MSCs and a group of animals with normal unscarred VFs was significantly different. These results suggest that most or all of the negative impact of scarring on the biomechanical properties of the VFs was ameliorated. However, this outcome was not always reported or measured.

### Mucosal wave amplitude after treatment of VF scarring with MSCs

Three studies reported measurements of the mucosal wave amplitude after induction of VF scarring. Table 4 describes these results. However, none of these studies reported statistics of the comparison between MSC-treated versus saline in scarred VFs. These studies only reported findings from the comparison between a combined approach of either MSC+SIS versus SIS/MSC or MSC + atelocollagen versus sham, which resulted in a statistically significant improvement versus a control group. None of the studies reported a measure of the magnitude of the difference between the MSC-treated versus saline-treated VFs. Therefore, it is difficult to evaluate whether the observed differences between the control group and the MSC-treated group were clinically relevant. However, a few of the studies reported a comparison between the MSC-treated group versus a group with unscarred VFs, and these studies did not find a significant difference between the MSC-treated group and the non-scarred group, which indicates that the intervention resulted in a relevant amelioration of mucosal wave function after intervention with MSCs.

**Table 4:** Study results from mucosal wave measurement.

Testing method	Author (year) [reference]	Time from wounding to intervention	Time from intervention to biomechanical testing	Comparisons	P-value
Mucosal wave measurement, videokymography	Choi et al (2014) [19]	0 days	8 weeks	MSC vs. MSC + SIS MSC vs. control	P < 0.01 Not reported
Mucosal wave measurement, videokymography	Kim et al (2014) [22]	0 days	1 and 3 months	MSC vs. PBS	Not reported
Mucosal wave measurement, high-speed digital-imaging	Ohno et al (2011) [23]	0 days	6 months	MSC + atelocollagen vs. sham	P < 0.01

### Use of MSCs for the treatment of acute or chronic vocal fold scarring

MSCs were injected into animal VFs on the same day as the scarring was performed in six of the seven studies. Therefore, these studies include the possibility of reducing scar tissue formation from the onset of healing, when a known insult to vocal fold tissue is performed. Only one study investigated the possibility of reducing already manifested scar tissue. The VFs in this model were scarred and left to heal with scarring for 9 weeks before surgically removing the scar tissue and injecting MSCs into the VFs during the same procedure. It is difficult to know in advance whether a given insult to human VFs will result in significant scar tissue formation. Therefore, this last model represents a more relevant model for the treatment of VF scarring using MSCs.

### Disease models used to induce scar tissue formation

Six of the studies used surgical instruments to induce scarring. However, the procedure was not described in detail regarding the extent of the insult, but most of the studies suggest that the entirety of the mucosa and lamina propria was removed. Some or most of the thyroarytenoid muscle was also surgically removed in four studies. Scar tissue formation was induced using an electrocoagulator in one study. The healing process during inflammatory disorders or after radiotherapy is different than surgically induced scars, but whether the treatment of scarred VFs after these types of insult would result in the same outcomes as these studies cannot be assessed.

### The persistence of the injected MSCs

The injected cells were detected at the evaluation time in four of the seven studies. Two of the seven studies detected no MSCs at the evaluation time, and no measurement of the persistence was performed in one study. Whether the presence or absence of the MSCs correlated to the effect of the injected stem cells is not known because of the largely unknown mechanism of action.

### Clinical trials evaluating the use of MSCs for the treatment of vocal fold scarring

The electronic search identified two on-going clinical trials, [26, 27] but none of the studies reported any preliminary data (Table 5). The first study is a phase I study used autologously expanded BM-MSC injections into patients with dysphonia and scarred vocal folds. The other study used adipose-derived SVFs that were directly injected into scarred vocal folds. Both studies evaluated biomechanical parameters of the vocal folds and voice quality.

**Table 5:** Overview of clinical trials.

Reference	Study type (n = estimated enrolment)	Intervention	Phase	Status	Outcome measures
Karolinska UH. [26]	Clinical, single-group, open labelled (n = 25)	Injection of autologous BM-MSC (aMSC) or aMSC with a hyaluronan gel in patients with vocal fold scarring	I	Recruiting	Safety, efficacy. Healing detection: inflammation, polyp/granuloma formation, blood sample evaluation. Functional measures: high-speed imaging, acoustic voice analysis, phonation pressure measurement.
Assistance PHDM. [27]	Clinical, single-group, open labelled (n = 8)	Injection of autologous stromal vascular fraction (SVF) in patients with vocal fold scarring	Not specified	Not yet recruiting	Feasibility, safety, efficacy. Functional measures: Voice handicap index evaluation, laryngostroboscopy

## Discussion

To the best of our knowledge, this study is the first systematic review of functional outcomes after mesenchymal stem cell treatment of VF scarring. No clinical trials have evaluated the effect of stem cell injections, and therefore, our results are based on preclinical animal studies. The use of MSCs in the treatment of vocal fold scarring is a complex research field, and several factors must be considered when evaluating the effect of MSC in the treatment of VF dysfunction after scarring. These factors include the type and dose of MSCs (adipose-derived or bone-marrow derived), the origin of MSCs (xenogeneic, allogeneic or autologous), the study design (acute or chronic scarring), and the disease model (surgery or electro coagulation to induce scarring). Three of the studies primarily examined a combined approach with MSCs and a type of scaffold or ECM protein, but these studies did not report the statistics of the comparison between an MSC-treated group versus saline-treated group although these experiments were performed. These methodological differences make it difficult to summarize the findings into a coherent conclusion. Potential biases in the studies were also a concern because only a few of the studies reported the randomization of animals into treated or control groups, and none specified how the randomization was performed. Only one study reported blinding.

Five of the seven studies evaluated the biomechanical properties of excised VFs, and most of these studies reported improvement in the MSC-treated group. However, not all of the studies reached statistical significance. Whether this difference in statistical significance was related to the low power used to detect a difference or methodological differences cannot be evaluated.

Three of the studies examined mucosal wave patterns after an intervention with MSCs, and most of these studies reported an apparent improvement. However, only one study reported the statistics (P < 0.01) for the comparison between the MSC- and saline-treated control groups. The significance of the comparisons in the two remaining studies was not reported.

Taken together, the above-mentioned findings are encouraging for further studies. There are some fundamental limitations in the animal studies that will make it difficult to evaluate whether human trials with MSCs will lead to clinically relevant improvements in phonation following an MSC treatment. First, six of the seven studies evaluated the reduction of scar tissue formation in the acute phase of healing. It is unlikely that this timeframe represents a relevant model of the treatment of human VF scarring. A relevant model would require the administration of MSCs during a planned insult to the VFs, which may be relevant in some, but not all clinical scenarios. It is unlikely that MSC treatment would be given before an observation period to assess any spontaneous improvement in phonation before offering any treatment because of the relative complexity of the current autologous MSC treatments. Only one study investigating the effect of MSC treatment of chronic scar tissue formation has been published, and this study reported encouraging improvements in biomechanical properties following MSC treatment. Studies comparing time perspectives of the injection of stem cells in relation to the specific trauma should be performed to obtain a treatment regimen focused on the exact cause of the VF lesion.

We identified one other review of vocal fold regeneration. This review primarily focused on histological changes following MSC treatment with various types of scaffolds and growth factors.[3] The correlation between histological changes and phonation is not straightforward. Therefore, we only focused on functional outcome measures, which exhibits a proven correlation with phonation, such as measures of a dysfunction, e.g., hoarseness and an easily fatigued voice. The mechanism of action is largely unknown, and further research on the exact effect of stem cell injection could clarify the results from the animal studies, such as histological changes and the persistence of the stem cells.

The results of the two uncompleted clinical trials, one using expanded MSCs and the other using the heterogeneic SVF cell population, should provide useful results in the field, especially for assessments of safety concerns and feasibility. However, the limited number of clinical studies indicates that much more research is necessary to provide evidence of MSC therapy for the treatment of scarred vocal folds. The authors are currently designing a prospective, blinded clinical trial of the injection of mesenchymal-derived stem cells perioperatively in Reinke’s edema patients.

In conclusion, animal studies of MSC treatment of scarred vocal folds reveal encouraging results for biomechanical and mucosal wave measurements. These results should be viewed cautiously with the limitations in the included studies, such as statistical insignificance, great intervention variance and the existence of potential biases. Whether these results will translate to improved phonation in human trials with MSC treatment for vocal fold scarring is currently not known. The results from the two on-going clinical trials with MSCs for VF dysfunction should answer some of these questions and are awaited with great interest.

## Supporting Information

S1 Appendix Search strategy appendix. (DOCX)

S1 File PRISMA 2009 checklist. (DOC)

S1 Table Supplementary table. (DOCX)
