# Supplementary material for: Mesenchymal Stem Cell Therapy for the Treatment of Vocal Fold Scarring: A Systematic Review of Preclinical Studies
Source: PLoS One. 2016 Sep 15;11(9):e0162349. doi: 10.1371/journal.pone.0162349 (PMC5025194; doi:10.1371/journal.pone.0162349)
Supplement: S1 Appendix — (DOCX) [file pone.0162349.s001.docx]

**Search strategy appendix**

*PubMed Advanced Search*: ("vocal folds" OR "vocal cords") AND ("stem cell" OR "MSC" OR "mesenchymal stem cell" OR "pre-adipocytes" OR "adipose stromal cell" OR "processed lipoaspirate cell" OR "stromal vascular fraction cell")

*Embase Advanced Search*: ("vocal folds" or "vocal cords") and ("stem cell" or "MSC" or "mesenchymal stem cell" or "pre-adipocytes" or "adipose stromal cell" or "processed lipoaspirate cell" or "stromal vascular fraction cell")

*Google Scholar Advanced Search*: allintitel: ("vocal folds" OR "vocal cords" OR "vocal fold" OR "vocal cord") ("stem cell" OR "stem cells" OR MSC)

*The Cochrane Library* (title, abstract, keyword): ("vocal folds" OR "vocal cords") AND ("stem cell" OR "MSC" OR "mesenchymal stem cell" OR "pre-adipocytes" OR "adipose stromal cell" OR "processed lipoaspirate cell" OR "stromal vascular fraction cell")

*ClinicalTrials*: ("vocal folds" OR "vocal cords") AND ("stem cell" OR "MSC")
