# Supplementary material for: Mesenchymal Stem Cell Therapy for the Treatment of Vocal Fold Scarring: A Systematic Review of Preclinical Studies
Source: PLoS One. 2016 Sep 15;11(9):e0162349. doi: 10.1371/journal.pone.0162349 (PMC5025194; doi:10.1371/journal.pone.0162349)
Supplement: S1 Table — (DOCX) [file pone.0162349.s003.docx]

**Supplementary table.**

| ***Outcome measures in preclinical studies*** | |
| --- | --- |
| **Macroscopic (via endoscopy)** | - Scar formation and wound healing |
| **Morphologic** | - Surface irregularities - Atrophic changes/volume reduction - Fibrous changes, granulation tissue or polyps - Regeneration |
| **Biomechanical/functional** | - Viscoelastic properties (via rheometry); dynamic viscosity and elastic modulus - Mucosal wave visualization (via. highspeed camera and vidoechymography) |
| **Microscopic; histologpathologic and immunehistochemic** | - ECM protein components; collagen staining, HA, elastin, decorin, fibronectin - Underlying muscle: smooth muscle actin - Fibrosis and ECM restoration/organization - Lamina propria thickness, polypformation - Inflammation; inflammatory cells |
| **Transcriptional** | - Enzyme up regulation - Growth factor up regulation (e.g. HGF and Fgf2, TGF-B) |
| **Detections of stem cells** | - Persistence - Proliferation - Apoptosis - Differentiation |
| **Side effects** | - Malignancy - Teratoma formation |
